# Neuroprotective and anti-inflammatory properties of proteins secreted by glial progenitor cells derived from human iPSCs

**DOI:** 10.3389/fncel.2024.1449063

**Published:** 2024-08-06

**Authors:** Diana I. Salikhova, Margarita O. Shedenkova, Anastasya K. Sudina, Ekaterina V. Belousova, Irina A. Krasilnikova, Anastasya A. Nekrasova, Zlata A. Nefedova, Daniil A. Frolov, Timur Kh. Fatkhudinov, Andrey V. Makarov, Alexander M. Surin, Kirill V. Savostyanov, Dmitry V. Goldshtein, Zanda V. Bakaeva

**Affiliations:** ^1^Laboratory of Cellular Biotechnology, Research Institute of Molecular and Cellular Medicine, Medical Institute of RUDN University, Moscow, Russia; ^2^Laboratory of Stem Cell Genetics, Research Centre for Medical Genetics, Moscow, Russia; ^3^Medical Genetic Center, National Medical Research Center for Children’s Health, Moscow, Russia; ^4^I.M. Sechenov First Moscow State Medical University (Sechenov University), Moscow, Russia; ^5^Institute of Information Technologies, MIREA-Russian Technological University, Moscow, Russia; ^6^Laboratory of Fundamental and Applied Problems of Pain, Institute of General Pathology and Pathophysiology, Moscow, Russia

**Keywords:** human induced pluripotent stem cells, glial progenitor cells, secreted proteins, glutamate excitotoxicity, LPS-induced inflammation

## Abstract

Currently, stem cells technology is an effective tool in regenerative medicine. Cell therapy is based on the use of stem/progenitor cells to repair or replace damaged tissues or organs. This approach can be used to treat various diseases, such as cardiovascular, neurological diseases, and injuries of various origins. The mechanisms of cell therapy therapeutic action are based on the integration of the graft into the damaged tissue (replacement effect) and the ability of cells to secrete biologically active molecules such as cytokines, growth factors and other signaling molecules that promote regeneration (paracrine effect). However, cell transplantation has a number of limitations due to cell transportation complexity and immune rejection. A potentially more effective therapy is using only paracrine factors released by stem cells. Secreted factors can positively affect the damaged tissue: promote forming new blood vessels, stimulate cell proliferation, and reduce inflammation and apoptosis. In this work, we have studied the anti-inflammatory and neuroprotective effects of proteins with a molecular weight below 100 kDa secreted by glial progenitor cells obtained from human induced pluripotent stem cells. Proteins secreted by glial progenitor cells exerted anti-inflammatory effects in a primary glial culture model of LPS-induced inflammation by reducing nitric oxide (NO) production through inhibition of inducible NO synthase (iNOS). At the same time, added secreted proteins neutralized the effect of glutamate, increasing the number of viable neurons to control values. This effect is a result of decreased level of intracellular calcium, which, at elevated concentrations, triggers apoptotic death of neurons. In addition, secreted proteins reduce mitochondrial depolarization caused by glutamate excitotoxicity and help maintain higher NADH levels. This therapy can be successfully introduced into clinical practice after additional preclinical studies, increasing the effectiveness of rehabilitation of patients with neurological diseases.

## 1 Introduction

Glutamate is a key neurotransmitter at the synapses responsible for transmitting nerve impulses in the brain. However, its excess amount can activate NMDA (N-methyl-D-aspartate) receptors, leading to the accumulation of excess amounts of intracellular calcium ions. Calcium ions play an important role in normal neuronal function, while excess amounts can cause enzyme activation, free radical production, and cell membrane destruction (Pinelis et al., 2022). These processes can lead to apoptosis (programmed cell death) and neuronal degeneration (Gleichmann and Mattson, 2011; Zündorf and Reiser, 2011). This process is called glutamate excitotoxicity and is associated with various neurological conditions such as ischemia, traumatic brain injury, and neurodegenerative diseases, including Parkinson’s disease and Alzheimer’s disease (Scorziello et al., 2020; Ge et al., 2022; Ludhiadch et al., 2022). Many neurological diseases are often accompanied by inflammatory processes, which play a key role in the pathogenesis of these conditions. These pathological processes lead microglia and astrocytes activation, which secrete cytokines, such as interleukins and tumor necrosis factor (TNFα), as well as neurotoxic molecules such as nitric oxide (NO) and reactive oxygen species (Witcher et al., 2021). NO synthesized by iNOS has diverse physiological functions. It may play a protective role in the immune response, while its excessive secretion can lead to negative consequences including hypoxia, oxidative stress and tissue damage. Inflammation can result in the blood-brain barrier disruption, increase the effects of inflammatory mediators on neural tissues, and exacerbate glutamate neurotoxic effects. There are studies that show the influence of pro-inflammatory cytokines, such as TNFα and IL-1β, on glutamate homeostasis in neural tissue. Thus, IL-1β can inhibit the uptake of this neurotransmitter by astrocytes, and TNFα can enhance the expression of AMPA receptor subunits and increase the concentration of glutamate in the synaptic cleft by inhibiting transporters on the presynaptic membranes of astrocytes, which leads to increased excitotoxicity effects (Zou and Crews, 2005; Ferguson et al., 2008). Dysregulation of cytokines, nitric oxide, and reactive oxygen species in neural tissue can trigger chronic inflammation, affecting neuronal survival and nervous system function. Recent studies have shown a link between neuroinflammation and the development of neurodegenerative diseases, 30% of people with traumatic brain injury having diffuse Aβ plaques in their brains (Djordjevic et al., 2016). Chronic inflammation has also been linked to the development of Parkinson’s disease. There are studies showing that activated microglia promote alpha-synuclein aggregates proliferation, resulting in damage to even more glial cells and neurons (Kasen et al., 2022). Moreover, proinflammatory cytokines, nitric oxide, and reactive oxygen species released from activated microglia can induce Ser129 phosphorylation of α-synuclein by activating protein kinase R, which is assumed to be pathologically significant (Reimer et al., 2018; Kawahata et al., 2022).

Treatment of many neurological diseases associated with inflammation and glutamate dysregulation includes the use of anti-inflammatory drugs, immunomodulatory agents, and NMDA receptor antagonists. However, many drugs have not yet passed clinical trials. For example, the negative side effects of MK-801 led to the abandonment of the use of NMDA receptor antagonists, since these receptors play an important role in glutamatergic system functioning (Janus et al., 2023). New research directions include the use of bioactive progenitor cell molecules aiming at specific molecular targets (Huang et al., 2020; Shalaby and El-Agnaf, 2022; Palanisamy et al., 2023). It is known that glial cells provide trophic support to neurons, releasing various physiologically active substances, and also influence the formation of synapses and neural networks (Lee et al., 2022; Valles et al., 2023). However, the mechanisms of the neuroprotective and anti-inflammatory effects of glial cells are poorly understood.

This study aims to investigate the anti-inflammatory and neuroprotective effects of proteins secreted by glial progenitor cells (SP-GPCs) derived from human induced pluripotent stem cells (iPSCs) in a model of LPS-induced inflammation and glutamate excitotoxicity.

## 2 Materials and methods

### 2.1 Preparation of secreted GPCs proteins

Cultures of glial progenitor cells (GPCs) were used for obtaining secretory proteins. GPCs were previously obtained from iPSCs, i.e., the “CTS CytoTune-iPS 2.1 Sendai Reprogramming Kit” (Invitrogen, USA) was used to reprogram dermal fibroblasts (Salikhova et al., 2021). Taking a skin biopsy from donors was approved by the Institutional Ethics Committee of the Research Centre for Medical Genetics (Protocol No. 2019-2/3 from October 13, 2020). Differentiation of iPSCs in the glial direction (GPCs) was carried out in two stages. At the first stage iPSCs were cultured in DMEM/F12 medium (Gibco, USA) with the addition of small molecules SB431542 (Stemcell Technologies, USA), 2 μM dorsomorphin (Stemcell Technologies, USA), and 200 nM LDN193189 (Sigma-Aldrich, USA). Then the cells were cultivated in DMEM/F12 medium supplemented with 10 ng/ml fibroblast growth factor (FGF-2), 20 ng/ml epidermal growth factor (EGF) (ProSpec, UK) and 20 ng/ml ciliary neurotrophic factor (CNTF) (PeproTech, USA) until spindle-shaped morphology cells were formed. Matrigel (Corning, USA) was used as a substrate. To obtain a conditioned medium, the GPCs were washed with Hanks’ solution and the following medium was added: DMEM/F12, 15 mM HEPES, 1 mM glutamine, 1% mixture of essential amino acids, 100 mg/l penicillin-streptomycin (all reagents - PanEko, Russia). GPCs were cultured in this medium for 16 hours. Further, the resulting conditioned medium (CM) was collected and centrifuged for 5 minutes at 3,000 rpm to remove cell debris. After this, the CM was passed through 0.45 μm and 0.22 μm filters on a vacuum unit (Millipore, Germany). Then, tangential ultrafiltration was performed on a Labscale TFF System (Millipore, Germany) using a Pellicon XL 100 kDa cartridge to remove proteins above 100 kDa. Then the membrane on the cartridge was changed to Pellicon XL 5 kDa and sequential concentration was carried out. The DMEM/F12 medium was replaced with phosphate-buffered saline (PBS) using the diafiltration mode. Using Amicon Ultra filters (Millipore, Germany), proteins were concentrated in PBS to 250 μg/ml. The resulting solution was aliquoted in 30 μl increments and stored at −80°C. Polyacrylamide gel electrophoresis was performed to check the molecular weight of secretory proteins. According to electrophoresis, proteins had a molecular mass predominantly less than 100 kDa, with a pronounced band around 25 kDa (Salikhova et al., 2023).

### 2.2 Measuring protein concentration using the Bradford method

For each sample, protein concentration was measured using the Bradford method with the help of the Quick Start Bradford protein assay kit (Bio-Rad Laboratories, USA). Concentration measurements were carried out according to a standard protocol; bovine serum albumin was used as a calibration standard (Kruger, 2009).

### 2.3 Ethical principles and regulatory documents

Experiments with animals were performed in accordance with the ethical principles and regulatory documents recommended by the European Science Foundation (ESF) and the Declaration on Humane Treatment of Animals and in accordance with the Order of the Ministry of Health and Social Development of Russia No 708n of August 23, 2010 “On Approval of Laboratory Practices”. Animal care, breeding and experimental procedures were carried out as required by the Ethical committee of the Institute of General Pathology and Pathophysiology. Protocol No 05-06/12 of 14.12.2017. For collection skin biopsy from donors, informed consent has been obtained from all patients involved in the study. The procedure was approved by the Institutional Ethics Committee of the Research Centre for Medical Genetics (Protocol No. 2019-2/3 from 13 October 2020).

### 2.4 Preparation of a primary cortical neuronal mixed culture

Wistar rat pups (P1-P2) were used for preparation of a primary mixed neuronal and glial culture of the cerebral cortex (primary neuroglial cultures) as previously described (Bakaeva et al., 2022). Breafly, the animals were anesthetized, decapitated, and the brain was removed. The cerebral cortex was isolated and cleaned from the blood vessel lining. A suspension of cortical neuroglial cells (10^6^ cells/ml) was obtained by treating brain tissue with papain (10 units / ml), and then dissociated by pipetting, the cells debris was washed out by sedimentation in a centrifuge (200g) in a Ca^2+^-free solution, then in Ca^2+^-containing solution and finally in the neurobasal medium (NBM, “Gibco”, USA). Cells were seeded in 48-well flat-bottom plates (2.5*10^5^ cells/well) (“Costar”, USA) and in Petri dishes (Ø35 mm) with a glass insert (Ø14 mm) at the bottom (“MatTeck”, USA). Plates and Petri dishes were pre-coated with polyethyleneimine (0.05 mg/ml, 60 min). One hour later, 1.5 ml of NBM containing 2% Supplement B-27, 1% Glutamax and 1% an antibiotics/antimycotic mixture (Gibco reagents, USA) were added. The cells were incubated within 10–14 days in vitro (DIV) at 37°C in an atmosphere of 5% CO2/95% air at 100% humidity. The medium was changed every three days, 1/3 of the medium replaced with fresh one. The mature mixed neuroglial culture contained predominantly cortical neurons. To get the immunophenotypic characterization of the resulting primary culture, an immunocytochemical test for the neuronal marker β-III-tubulin and the astrocytic marker of glial fibrillary acidic protein (GFAP) was carried out.

### 2.5 Obtaining primary mixed microglia culture

Primary microglia culture with an admixture of astrocytes was obtained from the cerebral cortex of Wistar rat pups (P1) (Lian et al., 2016; Bohlen et al., 2019). The extracted cerebral cortex was washed 2–3 times in Hanks’ solution containing 10% HEPES (PanEco, RF), crushed and transferred to a 0.05% trypsin solution with 0.02% EDTA (PanEco, RF) preheated to 37°C. After a 15-minute incubation, the cell suspension was washed twice with Hanks’ solution (PanEko, RF). The cells were disaggregated to obtain a homogeneous suspension, which was centrifuged for 2.5 min at 3,000 rpm. The cell sediment was resuspended in an appropriate volume of DMEM/F12 culture medium containing 10% serum (Gibco, USA), 1 mM glutamine (PanEco, Russia), 1% mixture of essential amino acids (PanEco, Russia), and 100 mg/l penicillin-streptomycin (PanEco, Russia). The cell suspension was transferred into T75 culture flasks (SPL, South Korea), pre-coated with Matrigel (Corning, USA). The primary culture was incubated for 6–7 days to achieve 80–90% confluency. To get the immunophenotypic characterization of the resulting culture an immunocytochemical test for the astrocytic marker of glial fibrillary acidic protein (GFAP) and the microglial marker CD11b was carried out.

### 2.6 Immunocytochemical analysis

For immunocytochemical analysis, cells were fixed with 4% formaldehyde solution (Merck KGaA, Germany) for 10 minutes at room temperature and permeabilized with 0.25% Triton X-100 solution (Sigma-Aldrich, USA) for 30 minutes. Then they were incubated overnight at +4°C with primary anti-iNOS antibodies (1:500, ab178945 Abcam), β-III-tubulin TUBB3 (1:500, ab78078 Abcam), glial fibrillary acidic protein GFAP (1:600, ab7260 Abcam), and CD11b (1:400, ab1211 Abcam). To visualize the expression of the listed proteins, cells were incubated in the dark for 60 minutes with anti-mouse secondary antibodies Alexa Fluor 555 (1:600) or anti-rabbit secondary antibodies Alexa Fluor 488 (1:600) (Invitrogen, USA). Nuclei were stained with a solution of DAPI (4,6-diamidino-2-phenylindole dihydrochloride; Sigma-Aldrich, USA) 1 μg/ml in phosphate-buffered saline (Salikhova et al., 2021). Images were obtained using an inverted fluorescence microscope Axio Observer.D1 with an AxioCam HRc camera (Carl Zeiss, Germany). For quantitative analysis of iNOS-positive cells, fluorescence intensity was measured on a plate reader (PerkinElmer, USA) by exciting fluorescence at 495 nm and recording emission at 519 nm.

### 2.7 Model of glutamate excitotoxicity

To model glutamate excitotoxicity, we used a primary culture of cortical neurons cultured in 48-well plates (Corning, USA) in an amount of 2*10^5^ cells/well. At 10 DIV, SP-GPCs were added at concentrations 5, 15, and 45 μg/ml. The next day, glutamate excitotoxicity was modeled. For this purpose, the culture medium was removed and the cortical neurons culture was washed with a DPBS solution without Ca^2+^ and Mg^2+^ (ThermoFisher, USA). Next, the cells were incubated for 1 hour in a buffer solution of the following composition (mM): NaCl−140, KCl−5, CaCl_2_−2, glycine−10, HEPES−20, glucose−5 (pH 7.4) with glutamate addition (100 μM) (all reagents - Sigma-Aldrich, USA). After that, the cortical neurons culture was washed twice in a buffer containing (mM): NaCl−140, KCl−5, MgCl_2_−2, HEPES−20, glucose−5 (pH 7.4) (Sigma-Aldrich, USA) and the conditioned culture medium was returned (Zgodova et al., 2022). A solution containing memantine as a voltage-dependent noncompetitive antagonist of NMDA-type glutamate receptors (Parsons et al., 2007) was added together with the glutamate solution (100 μM) and used as a positive control.

### 2.8 Assessing survival by biochemical method (MTT test)

Survival was assessed the next day after modeling glutamate excitotoxicity using the MTT test. MTT (3-[4,5dimethylthiazol-2-thiazolyl]-2,5-diphenyl-tetrazolium bromide, Merck, Germany) was added to the cells with the culture medium at a concentration of 0.1 mg/ml and incubated for 1 hour at 37°C. The culture medium was then removed and DMSO (ThermoFisher, USA) was added to dissolve formazan, a reduced tetrazolium salt (Ciapetti et al., 1993; Zgodova et al., 2022). The optical density of the solution was measured using a ClarioStars multimodal plate reader (BMG Labtech, Germany) at a wavelength of 520 nm and a reference wavelength of 690 nm. The obtained data were normalized by taking the absorption of formazan in control cultures as 100%.

### 2.9 Measurement of [Ca^2+^]i, mitochondrial potential ΔΨm and NADH in cortical neurons

Fluorescence microscopic measurements were carried out on the experimental setup involving an Olympus IX-71 inverted microscope equipped with 20 × and 40 × fluorite objectives, a Sutter Labmda 10-2 illumination system with a 175 W xenon lamp (Sutter Instruments, USA) and a CoolSNAP HQ2 CCD camera (Photometrics, USA), controlled through the MetaFluor computer program (Universal Imaging Corp., USA).

Measurements of the intracellular concentration of free Ca^2+^ ions ([Ca^2+^]_i_) and the transmembrane potential of mitochondria (ΔΨm) were carried out on 9-10 DIV cortical neurons, which were cultured on Ø35 mm glass bottom culture dishes (MatTeck, Ashland, MA, USA) at a concentration of 0.25*10^6^ as described earlier (Bakaeva et al., 2022). On day 10 after seeding cortical neurons, SP-GPCs were added at a concentration of 15 μg/ml, 24 hours before fluorescence microscopic measurements.

[Ca^2+^]_i_ measurements were performed using the low affinity fluorescent Ca^2+^ indicator Fura-2 in the form of acetoxymethyl ether (AM form) (Thermo Fisher Scientific, Waltham, MA, USA) at a concentration of 4 μM, by incubation in culture medium for 60 min at 37° C and 5% CO2. To facilitate the penetration of Fura-2 into cells, it was added in the form of a suspension with nonionic detergent Pluronik F-127 (0.02%. Sigma, Japan). Fura-2 fluorescence was excited alternately at 340 and 380 nm and recorded at 525 ± 25 nm (500 nm dichroic mirror). To simultaneously monitor changes in [Ca^2+^]_i_ and ΔΨm, cells were stained with the sensitive dye Rhodamine123 (Rh123, 2.5 μg/ml), (Thermo Fisher Scientific, Waltham, MA, USA) for 15 min. at a temperature of 37°C, the fluorescence of which was excited and recorded at 485 ± 5 and 525 ± 25 nm, respectively.

Measurements were carried out at 24–26°C in a buffer containing 130 mM NaCl, 5 mM KCl, 2 mM CaCl_2_, 1 mM MgCl_2_, 20 mM HEPES and 5 mM glucose (pH 7.4). Glutamate (100 μM) was introduced with glycine (10 μM) in a buffer without the addition of magnesium salts (MgCl_2_). After 15 minutes of exposure to glutamate, the cultures were incubated for 30 minutes in a nominally calcium-free buffer of the following composition: 130 mM NaCl, 5 mM KCl, 2 mM MgCl_2_, 20 mM HEPES, 5 mM glucose, 10 μM glycine, 100 μM EGTA (pH 7.4). The protonophore carbonyl cyanide-4-(trifluoromethoxy) phenylhydrazone (FCCP) (1 μM, 5 minutes in nominally calcium-free buffer) was then added to the cells to assess the maximum values of the Rh123 signal during mitochondrial depolarization and to assess the amount of Ca^2+^ accumulated by mitochondria. To determine the maximum cytoplasmic calcium capacity, the ionophore 1 μM ionomycin was used (in the presence of Ca^2+^ 5mM and without the addition of Mg^2+^).

Simultaneus measurements of the intracellular concentration NADH ([NADH]_i_) and ΔΨm were carried out on 14 DIV cortical neurons. Simultaneous measurements of changes in ΔΨm and intracellular NADH concentration were performed using Rh123 (Bakaeva et al., 2022) and NADH-dependent cell autofluorescence (Bartolomé and Abramov, 2015). The latter was excited and recorded at 365 ± 5 and 460 ± 25 nm, respectively, with a TFT-440-500-580 beam splitting mirror. Light filters and di- and trichroic mirrors are manufactured by Chroma (USA).

Data were acquired using MetaFluor software as images of the entire observed area and as the Microsoft Excel table for individually selected areas. The recorded images were processed in MetaFluor Analyst software (Universal Imaging Corp., USA).

### 2.10 Model of LPS-induced inflammation

To reproduce the inflammation model, a primary mixed culture of microglia and astrocytes was seeded onto 96-well plates (Corning, USA) in an amount of 3*10^4^ cells/well. To stimulate inflammation, lipopolysaccharide (Lipopolysaccharides from Escherichia coli, 500 ng/ml) (Sigma-Aldrich, USA) was added to the primary culture of astrocytes 10–12 DIV and incubated for 24 hours. At the same time, dexamethasone (0.6 μM) or secretory proteins (5, 15, 45 μg/ml) were added. Also, these concentrations of SP-GPCs were incubated with cultures not treated with LPS to determine the possible effect of proteins on NO secretion. Then the NO concentration was analyzed using the Griess reaction (Lieb et al., 2003; Paumier et al., 2022).

### 2.11 Griess test

To determine the NO concentration in a mixed culture of microglia and astrocytes, supernatants were collected. To the resulting supernatants (100 μl), pre-centrifuged for 5 minutes at 400 g, 100 μl of a 10% solution of Griess reagent in 12% acetic acid (LenReaktiv, Russia) was added. After a 15-minute incubation, the optical density was measured at 540 nm using a microplate reader (BioRad, USA). The values of the group with the addition of LPS were taken as 100% (Guevara et al., 1998).

### 2.12 Statistical data processing

All experiments were performed in duplicate at least 4 times on non-sister cell cultures. Statistical analysis was performed using GraphPad Prizm 8 (GraphPad Software, San Diego, CA, USA). Normality of distribution was assessed using the Shapiro-Wilk test. Data from the Griess test, MTT test and quantitative data from iNOS-positive cells were analyzed using one-way ANOVA with the Holm-Šidak correction; if the data distribution differed from the normal distribution, ANOVA on ranks was used with subsequent processing by Dunn’s test. Calcium imaging, ΔΨm, and [NADH]_i_ data were analyzed using a nonparametric t test followed by the Mann Whitney test. Data are presented as means and standard deviations or medians and interquartile ranges. Differences were considered statistically significant at a confidence level of p ≤ 0.05.

## 3 Results

### 3.1 Characteristics of a primary mixed culture of cortical neurons and assessment of the neuroprotective effect of SP-GPCs

**FIGURE 1:**
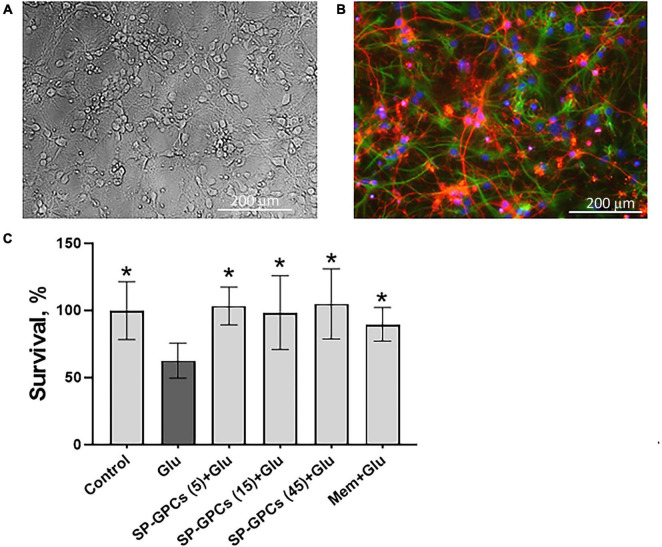
Characterization of cultured cortical neurons and assessment of dose-dependent effects of SP-GPCs in a glutamate excitotoxicity model. **(A)** Morphology of the resulting culture of cortical neurons. Phase contrast microscopy. **(B)** Immunophenotyping of neuroglial culture for astroglial GFAP (green) and neuronal β-III-tubulin (red) markers. Cell nucleus stained by DAPI (blue). Scale bar: 200 μm. **(C)** Determination of the viability of cortical neurons (MTT test); *p ≤ 0.05 compared to glutamate (Glu, 100 μM) (*n* = 5). Data were analyzed by one-way ANOVA with Holm-Šidak correction. Data are presented as means and standard deviations. SP-GPCs at concentrations from 5 to 45 μg/ml led to an increase in the number of viable neurons to control values. The addition of memantine (Mem, 100 μM) increased neuronal survival by 30 ± 13%.

On day 10 of cultivation (DIV), a mature mixed neuroglial culture with a predominant number of cortical neurons was obtained (Figure 1A). Morphologically, the neurons were distinguishable from glia and were round in shape with long neurites, laying in a focal plane above the glia, and were immunopositive for the neuronal marker β-III-tubulin (Figure 1B). The glial cells had an irregular contour with shorter processes and were immunopositive for the astrocytic marker of glial fibrillary acidic protein (GFAP) (Figure 1B). This type of culture was used to model glutamate excitotoxicity and measure intracellular parameters [Ca^2+^]_i_, ΔΨm and NADH.

24 hours after incubation of rat cortical neurons in the presence of 100 μM glutamate, a significant decrease in the number of living neurons was observed−to 62.6 ± 13% compared to the control (100%) (Figure 1C). In this case, preincubation of cultured neurons for 24 hours with SP-GPCs at concentrations from 5 to 45 μg/ml led to an increase in the number of viable neurons to control values. The addition of memantine (Mem, 100 μM) increased neuronal survival by 30 ± 13%.

### 3.2 Measurement of intracellular Ca^2+^ concentration ([Ca^2+^]i and mitochondrial transmembrane potential (ΔΨm)

**FIGURE 2:**
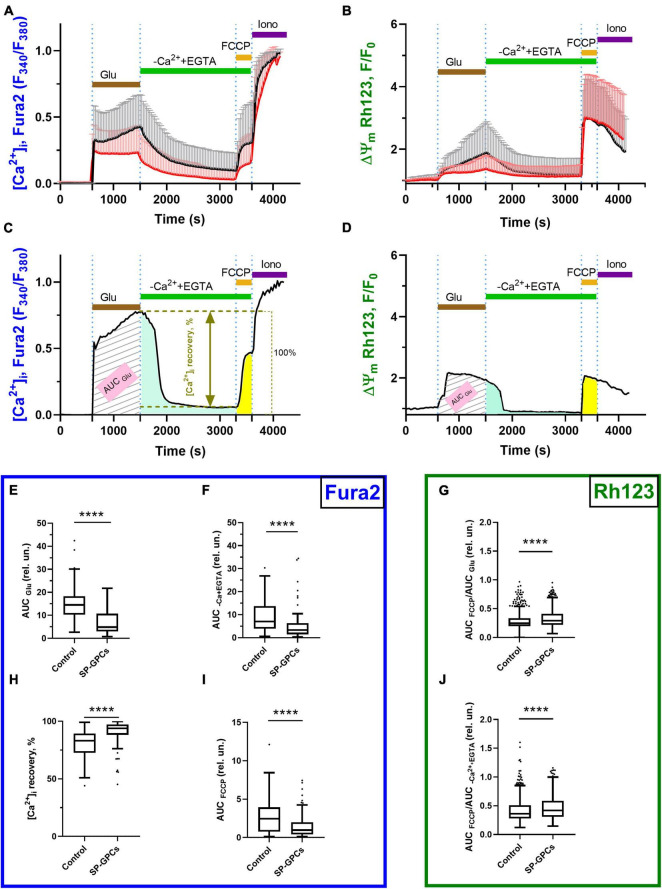
Comparison of parameters of dynamic changes in calcium homeostasis when modeling glutamate excitotoxicity under control and with the addition of SP-GPCs (15 μg/ml) 24 h before the experiment. [Ca^2+^]_i_ and ΔΨm measured using Fura2 and Rh123, respectively.**(A)** Average curves of [Ca^2+^]_i_ changes in the control group (black line, *n* = 485) and the group with the addition of SP-GPCs (red line, *n* = 434). **(B)** Average curves of the dynamics of changes in ΔΨm in the control group and the group with the addition of SP-GPCs. Data are presented as means and standard deviations. **(C,D)** Analysis of changes in [Ca^2+^]_i_ and ΔΨm by calculating the areas under the curves (AUC) in different periods using the example of one representative neuron. **(E)** Effect of SP-GPCs on AUC values during the period of Glu exposure (15 min) (AUC_Glu_), **(F)** during the period after removal of Glu in a nominally calcium-free buffer (20 min) (AUC_EGTA_), and **(H)** % recovery of the original level [Ca^2+^]_i_. **(I)** under the influence of protonophore FCCP (5 min) (AUC_FCCP_), **(G)** the degree of decrease in ΔΨm, expressed as the ratio AUC_FCCP_/AUC_Glu_, **(J)** the degree of recovery of ΔΨm, expressed as the ratio AUC_FCCP_/AUC_EGTA_. Data were analyzed using a nonparametric unpaired t test followed by the Mann–Whitney test and presented as medians and interquartile range. ****p ≤ 0.0001 compared to control.

Addition of glutamate (Glu) at a concentration of 100 μM (15 min) significantly increased cytoplasmic calcium [Ca^2+^]_i_ and simultaneously caused a drop in ΔΨm in almost all cells (Figures 2A, B). To assess intracellular changes in the study groups, several parameters were analyzed as previously described (Bakaeva et al., 2022). Briefly, the magnitude of changes in [Ca^2+^]_i_ and ΔΨm in individual neurons was assessed by changes in the area under the curve (AUC) of fluorescence of the corresponding probes Fura-2 (Figure 2C) and Rh123 (Figure 2D).

The intensity of accumulation of calcium ions in the cytoplasm of cells against the background of the action of glutamate, assessed using the AUC_Glu_ parameter, significantly decreased from the control values of 14.5 relative units (Q25 = 10.3−Q75 = 18.2; n = 485) to 4.8 relative units (Q25 = 30.2−Q75 = 10.7; n = 434) against the background of preincubation of cells with SP-GPCs (Figure 2E).

Removal of glutamate in a nominally calcium-free buffer resulted in a decrease of [Ca^2+^]_i_ in the study groups. At the same time, the value of the AUC_Ca_^2+^_+EGTA_ parameter significantly decreased to 3.3 relative units (Q25 = 1.4−Q75 = 6.2; n = 434) in the group with preincubation of cells with SP-GPCs compared to the control 7.0 relative units (Q25 = 3.9−Q75 = 13.7; n = 485), which indicates a more intense removal of Ca^2+^ ions from the cytoplasm of cortical neurons in the post-glutamate period (Figure 2F). This is consistent with the lower rise in [Ca^2+^]_i_ upon addition of FCCP (Figure 2I). The percentage of restoration of the initial level of [Ca^2+^]_i_ during this period significantly increased against the background of SP-GPCs to 93.8% (Q25 = 88.0−Q75 = 97.3; n = 434) compared to the control 83.1% (Q25 = 72.5−Q75 = 89.3; n = 485) (Figure 2H).

Mitochondria are the main intracellular store of calcium ions in neurons, protecting the cytosol and nucleoplasm from Ca^2+^ overload (Bakaeva et al., 2022). Upon depolarization of the inner mitochondrial membrane caused by the addition of protonophore FCCP (1 μM), the electrophoretic retention of Ca^2+^ in the mitochondrial matrix ceases and Ca^2+^ is released into the cytoplasm, rapidly increasing [Ca^2+^]_i_ (Figure 2B). The addition of SP-GPCs resulted in less Ca^2+^ release from mitochondria (Figure 2I). The AUC_FCCP_ value significantly decreased from 2.4 relative units (Q25 = 0.7−Q75 = 3.9; n = 485) to 0.9 relative units (Q25 = 0.4−Q75 = 1.9; n = 434).

To assess changes in ΔΨm before and after glutamate exposure, as well as against the background of the action of FCCP protonophore, the signal intensity of the voltage-sensitive mitochondrial probe Rh123 was measured. AUC_Glu_ and AUC_Ca_^2+^_+EGTA_ were calculated from Rh123 probe fluorescence curves for individual cells as described above and shown in Figure 2D. Addition of Glu (100 μM) resulted in increased Rh123 signal, indicating mitochondrial depolarization (Figure 2B). The addition of FCCP resulted in maximum signal enhancement, since the Rh123 dye accumulated in the cytoplasm after a sharp drop in ΔΨm. To determine the ability of cells to maintain ΔΨm, the AUC_FCCP_/AUC_Glu_ and AUC_FCCP_/ AUC_Ca_^2+^_+EGTA_ ratios were measured. The greater the ratio of AUC_FCCP_ to the stages under study, the more Rh123 dye accumulated in mitochondria due to their lower depolarization. The addition of SP-GPCs led to a significant increase in the AUC_FCCP_/AUC_Glu_ ratio to 0.291 relative units (Q25 = 0.219−Q75 = 0.410; n = 434) compared to control 0.245 relative units (Q25 = 0.195−Q75 = 0.334; n = 485) (Figure 2G). The AUC_FCCP_/AUC_Ca_^2+^_+EGTA_ ratio was also higher against the background of SP-GPCs 0.419 relative units (Q25 = 0.309−Q75 = 0.587; n = 434) compared to the control 0.363 relative units (Q25 = 0.280−Q75 = 0.510; n = 485) (Figure 2J).

### 3.3 Measurement of intracellular NADH

NADH is a major substrate of the mitochondrial respiratory chain (Sharma et al., 2009; Mimaki et al., 2012). In neurons, the overwhelming majority of NADH ( > 70%) is localized in mitochondria, and therefore changes in the intracellular level of this coenzyme are an important indicator of the functional activity of mitochondria (Ying, 2007). The dynamics of changes in NADH concentration can be judged by the intensity of endogenous cell fluorescence, excited in the UV region of the spectrum at ∼360 nm and recorded at ∼460 nm (Bartolomé and Abramov, 2015).

**FIGURE 3:**
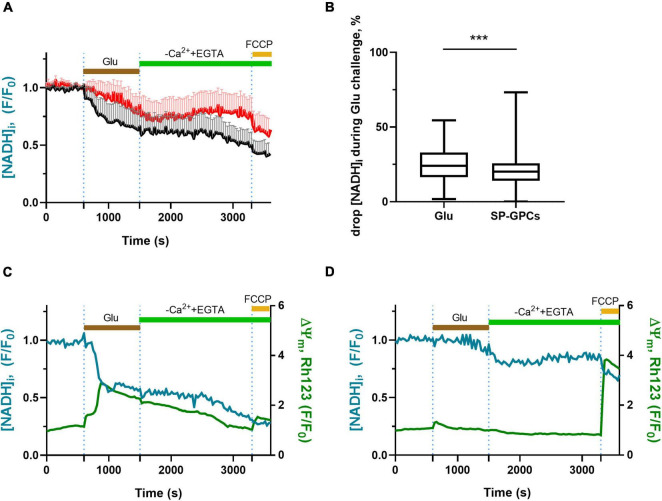
Effect of SP-GPCs on the dynamics of [NADH]_i_ changes under the influence of glutamate. Data are presented as means and standard deviations. **(A)** Average curves of changes in [NADH]_i_ in the control group and the group with added SP-GPCs (*n* = 196 in the control group and 182 in the SP-GPCs group). **(B)** Decrease in [NADH]_i_ during 15 min of exposure to glutamate, expressed in %, the initial level of [NADH]_i_ is taken as 100%, and the level of [NADH]_i_ under exposure to FCCP is taken as 0%. **(C,D)** change in [NADH]_i_ and ΔΨm in representative neurons from the control and SP-GPCs supplemented groups, respectively. Data were analyzed using a nonparametric unpaired t test followed by the Mann–Whitney test and presented as medians and interquartile ranges. ***p ≤ 0.001 compared to control.

In experiments on cortical neurons (14 DIV) measuring intracellular NADH (predominantly mitochondrial), SP-GPCs were found to reduce the Glu-induced drop in NADH (Figure 3A). On average, the drop in the level of intracellular NADH during a 15-minute exposure to glutamate in the group of cells treated with SP-GPCs was 20.1% (Q25 = 13.6−Q75 = 25.7; n = 182) and in the control group 24.0% (Q25 = 16.3−Q75 = 33.0; n = 196) (Figure 3B). The most dramatic decrease in NADH occurred with a pronounced drop in ΔΨm (change in Rh123 signal) in response to glutamate (Figure 3C). Changes in ΔΨm were on average less significant in the presence of SP-GPCs than when exposed to Glu alone (Figure 3D).

### 3.4 Evaluation of the anti-inflammatory effect of SP−GPCs

**FIGURE 4:**
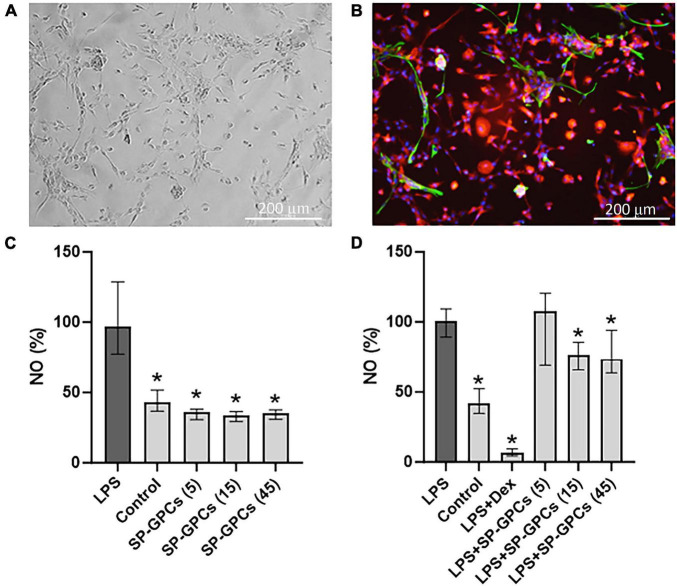
Characterization of mixed glial culture and assessment of the dose-dependent effects of SP-GPCs on the secretion of the inflammatory mediator NO. **(A)** Morphology of the resulting microglia culture with an admixture of astrocytes (10 DIV). Phase contrast microscopy. **(B)** Immunophenotyping of glial culture for astroglial GFAP (green) and microglial CD11b (red) markers. Cell nucleus stained by DAPI (blue). Scale bar: 200 μm. **(C)** relative NO secretion by astrocytes and microglia upon addition of SP-GPCs under physiological conditions, and **(D)** during LPS-dependent inflammation (Griess test). Data were analyzed by rank analysis of variance (ANOVA on ranks) followed by Dunn’s test and presented as medians and interquartile ranges, *p ≤ 0.05 compared with the LPS group, *n* = 5. In the model of LPS-dependent inflammation, the addition of SP-GPCs at concentrations of 15 and 45 μg/ml led to a decrease in NO secretion. NO values during incubation with SP-GPCs at a concentration of 5 μg/ml were comparable to those in the LPS group. As a positive control, dexamethasone (Dex; 0.6 μM) was supplemented.

At day 10 of culture (DIV), a mixed glial culture was obtained with a predominant number of microglia that were CD11b-immunopositive (Figures 4A, B). Also present in the culture were a few cells immunopositive for the astrocytic marker GFAP (Figure 4B). This type of culture was used to model an LPS-induced inflammation. NO is an important mediator of the inflammatory response and immune defense (Wink et al., 2011). Moreover, its excessive formation is associated with neurotoxicity and neuronal damage (Calabrese et al., 2007). The anti-inflammatory effect of SP-GPCs was assessed by reducing the concentration of NO in a primary culture of microglia and astrocytes under physiological conditions and in a model of LPS-dependent inflammation. Treatment of primary culture of microglia and astrocytes (10 DIV) with LPS (500 ng/ml, 24 hours) led to an increase in NO secretion to 96.86% (Q25 = 77.3−Q75 = 128.8; n = 5) compared to control 43.08 % (Q25 = 36.7−Q75 = 51.8; n = 5) (Figure 4C). Incubation with SP-GPCs did not lead to an increase in NO secretion by microglia and astrocytes under physiological conditions and was significantly different from the LPS group (Figure 4C). In the model of LPS-dependent inflammation, the addition of SP-GPCs at concentrations of 15 and 45 μg/ml led to a decrease in NO secretion, the values in these groups were 76.2% (Q25 = 65.8−Q75 = 85.4; n = 5) and 73.6 % (Q25 = 63.7−Q75 = 94.1; n = 5) (Figure 4D). NO values during incubation with SP-GPCs at a concentration of 5 μg/ml were comparable to those in the LPS group. As a positive control, dexamethasone (Dex; 0.6 μM) was supplemented, which significantly reduced NO secretion to 6.5% (Q25 = 4.4−Q75 = 9.4; n = 5) (Figure 4D).

**FIGURE 5:**
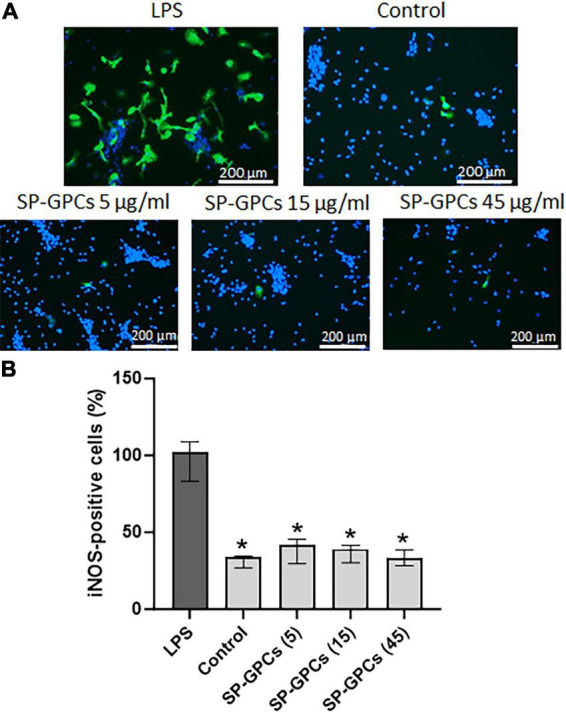
Inducible NO synthase activity upon addition of LPS and SP-GPCs to a mixed glial culture. **(A)** immunocytochemical study of iNOS (green cells). Cell nuclei are stained with DAPI (blue). **(B)** quantification of iNOS-positive cells, *n* = 4. Data were analyzed by rank analysis of variance (ANOVA on ranks) followed by Dunn’s test and presented as medians and interquartile ranges, *p ≤ 0.05 compared with LPS. Scale bar 200 μm. Incubation of a mixed glial culture (12 DIV) with LPS contributed to an increase in the number of iNOS-positive cells compared to the control. The addition of SP-GPCs at concentrations of 5 to 45 μg/ml under physiological conditions did not result in an increase in the population of iNOS-immunopositive cells.

iNOS is activated in response to inflammatory signals and can lead to a significant increase in NO synthesis in cells (Figure 5A). Incubation of a mixed glial culture (12 DIV) with LPS contributed to an increase in the number of iNOS-positive cells to 102.3% (Q25 = 83.3–Q75 = 109; n = 4) compared to the control 33.9% (Q25 = 26.8–Q75 = 34.6; n = 4) (Figure 5B). The addition of SP-GPCs under physiological conditions did not result in an increase in the population of iNOS-immunopositive cells (Figure 5B).

**FIGURE 6:**
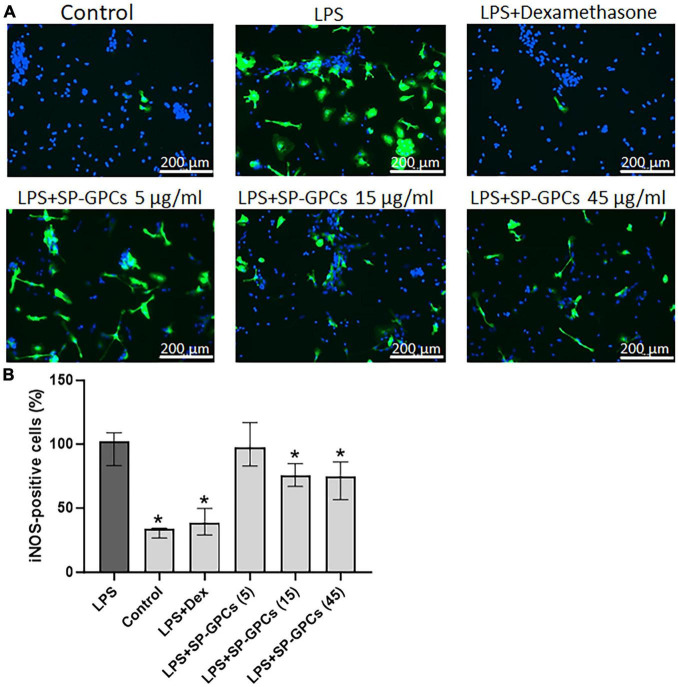
Inducible NO synthase activity upon addition of SP-GPCs to a mixed glial culture in a model of LPS-dependent inflammation. **(A)** immunocyto- chemical study of iNOS (green cells). Cell nuclei are stained with DAPI (blue). **(B)** quantification of iNOS-positive cells, *n* = 4. Data were analyzed by rank analysis of variance (ANOVA on ranks) followed by Dunn’s test and presented as medians and interquartile ranges, *p ≤ 0.05 compared with LPS. Scale bar 200 μm. In the model of LPS-dependent inflammation, the addition of SP-GPCs at concentrations of 15 and 45 μg/ml contributed to a decrease in iNOS-positive cells in a mixed glial culture (12 DIV). The addition of dexamethasone (Dex; 0.6 μM) during LPS-induced inflammation effectively reduced the number of iNOS-positive cells and led to comparable to control values.

In the model of LPS-dependent inflammation, the addition of SP-GPCs at concentrations of 15 and 45 μg/ml contributed to a decrease in iNOS-positive cells in a mixed glial culture (12 DIV) to 75.8% (Q25 = 26.8−Q75 = 34.6; n = 4) and 74.6 (Q25 = 56.7−Q75 = 86.1; n = 4), respectively (Figures 6A, B). The addition of dexamethasone (Dex; 0.6 μM) during LPS-induced inflammation effectively reduced the number of iNOS-positive cells to 38.7% (Q25 = 29.3−Q75 = 49.7; n = 4), which is comparable to control values (Figure 6B).

## 4 Discussion

Inflammation involving microglia and astrocytes, as well as excess accumulation of glutamate, is associated with many central nervous system (CNS) diseases, such as Alzheimer’s disease, Parkinson’s disease, ischemic stroke, and schizophrenia (Vesce et al., 2007). Astrocytes and microglia play a significant role in the regulation of NO production and secretion, which influences a variety of physiological and pathological processes (Fujita and Yamashita, 2021). NO produced by astrocytes may serve as a neuromodulator, affecting vascular regulation and neurotransmission (Kwon and Koh, 2020). iNOS in microglia is activated in response to inflammatory mediators, increasing NO levels, which can lead to oxidative stress and neuronal death (Tewari et al., 2020). Astrocytes are known to release a variety of active substances, including glutamate (Bezzi and Volterra, 2001; Angulo et al., 2004; Perea and Araque, 2007). Inflammatory processes in the brain stimulate the release of glutamate from activated astrocytes, microglial cells and other cells of the immune system, which can lead to excitotoxicity (Misra et al., 2020). Inflammation is also accompanied by the release of various cytokines and mediators that can modulate the function of glutamate receptors and metabotropic receptors, which in turn can also increase glutamate excitotoxicity (Fairless et al., 2021).

In the CNS, glial cells typically perform supportive functions, including participation in energy metabolism, synaptic plasticity, and ion homeostasis. In addition to providing support to neurons, astrocytes function as resident immune cells in the brain. Moreover, in response to local changes in extracellular Ca^2+^, they can strongly influence physiological and pathophysiological events in the nervous system (Sobolczyk and Boczek, 2022). Previously studies conducted, that SP-GPCs included proteins with anti-apoptotic function, such as 14-3-3 family, apolipoprotein A-I (APOA1), apolipoprotein D (APOD), gremlin (GREM1), serpin B3 (SERPINB3), and also proteins involved in the regulation of the immune response: antithrombin III (SERPINC1), galectin-1 (LGALS1), cathepsin D (CTSD) and macrophage migration inhibitory factor (MIF). SP-GPCs contained proteins belonging to the category of metabolic processes, such as reticulocalbin-1 (RCN1) is a calcium-binding protein, ataxin-1 (ATXN1), proteasome subunit beta (PSMB), peroxiredoxin-1 (PRDX1), and others (Salikhova et al., 2023). All listed protein has molecular weight less than 100 kDa.

In this work, using primary neuronal and glial cultures from the rat brain, the anti-inflammatory and neuroprotective effects of SP-GPCs were studied in a model of LPS-induced inflammation and glutamate excitotoxicity. It was shown that the addition of SP-GPCs neither led to activation of iNOS under standard cultivation conditions, nor enhanced NO synthesis. At the same time, concentrations of SP-GPCs of 15 and 45 μg/ml reduced the secretion of NO by astrocytes and microglia when modeling LPS-induced inflammation, which indicates proteins potential anti-inflammatory effect. This effect is explained by the inhibition of iNOS activity. Dexamethasone, used as a positive control for anti-inflammatory action, had a more pronounced effect than SP-GPCs. It is possible that SP-GPCs contain anti-inflammatory proteins, since a similar effect has previously been demonstrated in other cultures, in particular for multipotent mesenchymal stem cells (MMSCs) (Arabpour et al., 2021; Jin et al., 2022). In many studies, MMSCs suppressed iNOS expression and activity in macrophages and other immune cells by inhibiting NF-κB and other signaling pathways that regulate iNOS expression, thereby reducing NO production (Ooi et al., 2010; Holthaus et al., 2022; Sari et al., 2023). Rahmat et al. (2013) showed that MMSCs produced antioxidants such as superoxide dismutase (SOD) and glutathione, which neutralized free radicals and prevented oxidative stress, which in turn reduced iNOS activation and NO production (Rahmat et al., 2013). At the same time, the MMSC secretome decreased the expression of proinflammatory cytokines (IL-6, TNF-α) and increased the expression of anti-inflammatory factors (TGF-β, IL-10, IL-13) (Su et al., 2023). A number of studies have shown an increase in anti-inflammatory (M2) phenotype macrophages/microglia (Iba1^+^/Arg1^+^ cells) (Ferreira et al., 2018; Holopainen et al., 2020; Holthaus et al., 2022). This work demonstrates for the first time the effect of SP-GPCs on NO levels by reducing the activation of iNOS, which is consistent with the data of these studies.

In a model of glutamate excitotoxicity, the neuroprotective effect of SP-GPCs (5–45 μg/ml) was demonstrated. The addition of SP-GPCs led to an increase in the number of viable cells to control values, not being inferior in effectiveness to memantine, a non-competitive antagonist of NMDA receptors. The neuroprotective effect of SP-GPCs is due, firstly, to the ability to reduce the accumulation of calcium ions in the cytosol of cells when exposed to glutamate (100 μM), and secondly, to increase the efficiency of restoration of the initial level of [Ca^2+^]_i_ in the post-glutamate period. This effect is also mediated by the influence of SP-GPCs on the functional activity of mitochondria. We have previously shown that calcium dysregulation in neurons during glutamate excitotoxicity leads to pathological changes in mitochondria, such as loss of membrane potential and decreased ATP production, while substances that interfere with this can increase neuronal viability (Pinelis et al., 2022; Zgodova et al., 2022). It was shown that preincubation of SP-GPCs with cortical neurons promoted maintaining ΔΨm and higher NADH level. This is consistent with the lower rise in [Ca^2+^]_i_ upon Glu addition. It is likely that SP-GPCs contain components that reduce the Ca^2+^-conducting properties of NMDA receptors, since some studies previously demonstrated the possibility of inhibition of NMDA channel by certain peptides (Jimenez, 2022; Islam et al., 2023). Thus, SP-GPCs can reduce the need for cells to spend ATP to remove Ca^2+^ from the cell by Ca^2+^-ATPases and maintain the transmembrane potential of the plasmalemma by Na^+^/K^+^-ATPases. However, given that the cultures were incubated with SP-GPCs for 24 hours before exposure to glutamate, it cannot be excluded that the components of SP-GPCs may reduce the expression of NMDA receptors or reduce their cation-conducting properties due to changes in subunit composition. This hypothesis is supported by data from studies showing the neuroprotective effect of secreted proteins from stem/progenitor cells (Jha et al., 2021; Jacques et al., 2023).

Hao et al. (2014) demonstrated the neuroprotective effects of human adipose tissue-derived MMSCs (AMSCs) in a glutamate excitotoxicity model in cortical neurons (Hao et al., 2014). Conditioned medium of AMSCs (CM-AMSCs) partially reduced neuronal cell damage, as indicated by decreased lactate dehydrogenase (LDH) release and a decrease in the number of apoptotic cells. The authors believe this effect was associated with increased expression of GAP-43 and an increased number of GAP-43-positive neurites. In addition, incubation of neuronal cells with CM-AMSCs increased ATP, NAD^+^ and NADH levels and the NAD^+^/NADH ratio, while preventing glutamate-induced decrease in mitochondrial membrane potential. Another study showed that 24-hour preculture of primary mouse cortical neurons with MMSCs protected them from death caused by hyperactivation of NMDA receptors, and MMSC-derived conditioned medium (CM-MMSCs) was sufficient for this effect (Anda et al., 2012). Protection by CM-MMSCs was associated with reduced levels of mRNA genes encoding NMDA receptor subunits, as well as reduced glutamate-induced calcium ion influx (Anda et al., 2012). In addition, CM-MMSCs-mediated neuroprotection has been associated with an effect on the distribution of GluR1 AMPAR subunits on the surface of neuronal membranes, which also helps reduce neuronal death during glutamate toxicity (Papazian et al., 2018). There are also studies showing that CM-AMSCs reduce the death of spinal neurons during glutamate toxicity. The authors attribute this neuroprotective effect to a decrease in the positive regulation of apoptotic caspase-3 activation, as well as an increase in the expression of the anti-apoptotic protein gene Bcl-2 (Lu et al., 2015).

There is evidence that conditioned media from stem cells affects not only neurons, but also astrocytes. Gui et al. (2009) showed that pretreatment of astrocyte culture with bone marrow stromal cell conditioned media enhanced astrocyte glutamate uptake in a model of glutamate excitotoxicity (Gui et al., 2009). This work demonstrates for the first time the effect of SP-GPCs on increasing the survival of neurons by enhancing cellular metabolism and increasing the efficiency of regulation of calcium homeostasis during glutamate excitotoxicity, which is consistent with the data of the above-described works.

Thus, SP-GPCs have both neuroprotective and anti-inflammatory effects. In this regard, the question arises about the choice of cell type both for direct cell therapy and for obtaining CM. GPCs probably have more favorable biological properties due to the ability to secrete factors capable of supporting neurons. Based on the current study, it can be assumed that the GPCs culture will be a promising material for autologous transplantations, since it can be obtained from the patients themselves, and can also be used as a source of biologically active substances.

## 5 Conclusion

This study demonstrated anti-inflammatory and neuroprotective effects of proteins with a molecular weight below 100 kDa secreted by glial progenitor cells derived from human induced pluripotent stem cells. SP-GPCs decline inflammation caused by LPS due to reducing nitric oxide (NO) production through inhibition of iNOS. At the same time, they had the ability to increase neurons viability by neutralization the effect of glutamate excitotoxicity. Such effect associated with stabilization of mitochondrial membrane potential, maintain higher NADH levels, and lowering levels of intracellular calcium, an excessive amount of which leads to the death of neurons.

## Data availability statement

The original contributions presented in this study are included in this article/supplementary material, further inquiries can be directed to the corresponding authors.

## Ethics statement

The studies involving humans were approved by the Research Centre for medical genetics. The studies were conducted in accordance with the local legislation and institutional requirements. The participants provided their written informed consent to participate in this study. The animal study was approved by the Institute of General Pathology and Pathophysiology. The study was conducted in accordance with the local legislation and institutional requirements.

## Author contributions

DS: Conceptualization, Data curation, Visualization, Writing−original draft, Writing−review and editing. MS: Investigation, Methodology, Writing−review and editing. AKS: Investigation, Writing−review and editing. EB: Investigation, Writing−review and editing. IK: Software, Validation, Visualization, Writing−review and editing. AN: Investigation, Writing−review and editing. ZN: Investigation, Writing−review and editing. DF: Software, Validation, Visualization, Writing−review and editing. TF: Writing−review and editing. AM: Writing−review and editing. AMS: Data curation, Writing−review and editing. KS: Funding acquisition, Project administration, Writing−review and editing. DG: Conceptualization, Writing−original draft, Writing−review and editing. ZB: Conceptualization, Software, Validation, Visualization, Writing−original draft, Writing−review and editing.
